# Supplementary material for: Deep learning-based prediction of early cerebrovascular events after transcatheter aortic valve replacement
Source: Sci Rep. 2021 Sep 21;11:18754. doi: 10.1038/s41598-021-98265-5 (PMC8455675; doi:10.1038/s41598-021-98265-5)
Supplement: Supplementary file 1 — Supplementary Information. [file 41598_2021_98265_MOESM1_ESM.pdf]

## SUPPLEMENTAL MATERIALS

# Deep Learning-based Prediction of Early Cerebrovascular Events after Transcatheter Aortic Valve Replacement

*Okuno et al. Deep learning-based stroke prediction for TAVR*


Taishi Okuno, MD[a*]; Pavel Overtchouk, MD[a,b*]; Masahiko Asami, MD[a]; Daijiro Tomii, MD[a]; Stefan Stortecky, MD[a]; Fabien Praz, MD[a]; Jonas Lanz, MD[a]; George CM Siontis, MD[a]; Christoph Gräni, MD[a]; Stephan Windecker, MD[a]; Thomas Pilgrim, MD[a]

a) Department of Cardiology, Inselspital, University of Bern, Bern, Switzerland

b) AlvissLabs Research, ALVISS.AI SAS, 67 rue Saint-Jacques 75005 Paris, France

*Dr. Okuno and Dr. Overtchouk contributed equally to this work and are joint first authors.

**Corresponding Author:**

Pavel Overtchouk, MD

AlvissLabs Research

ALVISS.AI

75005 Paris

France

Phone: +31 7 50 05 48 31

Mail: pavel@alviss.ai

## TABLE OF CONTENTS

**SUPPLEMENTARY TABLE 1. 30-DAY PREDICTIVE MODEL DESCRIPTION .......................3**

**SUPPLEMENTARY TABLE 2. FULL LIST OF PREDICTIVE VARIABLES INCLUDED IN THE AUTOENCODER MODEL RANKED BY THEIR IMPORTANCE ............................................4**

**SUPPLEMENTARY FIGURE 1. PRECISION-RECALL CURVE FOR THE CHOICE OF DECISION THRESHOLD VALUE ..........................................................................................5**

**SUPPLEMENTARY FIGURE 2. TOP 20 VARIABLES CLASSIFIED BY IMPORTANCE FOR THE PREDICTIVE MODEL .........................................................................................7**

**Supplementary Table 1. 30-day predictive model description**

| Layer (type) | Output Shape | Param # |
|---|---|---|
| input_157 (InputLayer) | [(None, 157)] | 0 |
| dense_788 (Dense) | (None, 252) | 39816 |
| dense_789 (Dense) | (None, 126) | 31878 |
| dense_790 (Dense) | (None, 126) | 16002 |
| dense_791 (Dense) | (None, 252) | 32004 |
| dense_792 (Dense) | (None, 157) | 39721 |

Total params: 159,421
Trainable params: 159,421
Non-trainable params: 0

The model is an autoencoder which includes 159421 trained parameters. Learning rate 0.001, 200 epochs, batch size of 256, Adam optimizer, mean squared error as loss parameter and accuracy as optimization parameter.

**Supplementary Table 2. Full list of predictive variables included in the autoencoder model ranked by their importance**

| |
|---|
| History of valvuloplasty; Hematoma (complication); Clopidogrel pre-treatment; Ventricular perforation (complication); Reposition; Main access; Instability requiring ECMO (complication); Fistula (complication); Instability requiring assistance (complication); Aortic dissection (complication); >1 THV required; Left coronary height (CT); Aortic annulus perimeter (CT); Concomitant LAA or PFO closure; Post-TAVR aortic regurgitation grade (angiography); Conversion to SAVR (complication); Access site closure failure (complication); Aspirin pre-treatment; Non-coronary cusp DLZ calcification volume (CT); Shock (complication); Other access; Non-coronary cusp aortic valve calcification volume (CT); Ascending aorta diameter (CT); Peripheral artery disease; Calcium antagonist premedication; Diuretic premedication; Concomitant iliofemoral stenting (procedure); Contrast volume; Aortic valve calcification volume (CT); Canadian Cardiovascular Society grade of angina pectoris; Procedure location (hybrid or not); Chronic obstructive pulmonary disease; Concomitant PCI (procedure); Femoral stenting (complication); P2Y12 premedication; STS score; History of stroke (hemorrhagic and ischemic); Annulus area (CT); Aortic angulation (CT); Left coronary cusp valve calcification volume (CT); History of myocardial infarction; Tamponade (complication); Vascular access stenosis (complication); History of cerebral transient ischemic attack; Poly-medication; Anesthesia type; Type of aortic valve anatomy (CT); Porcelain aorta (CT); Total DLZ calcification volume (CT); Mitral regurgitation before TAVR (echocardiography); Complication requiring surgery (complication); new-onset atrial fibrillation (complication); Anti-thrombotic management before TAVR; Left coronary cusp DLZ calcification volume (CT); Balloon dilatation (procedure); Aortic regurgitation before TAVR (echocardiography); Location of raphe (CT); Right coronary cusp DLZ calcification volume (CT); Aortic valve area (echocardiography); Sheath size; Age; Heart rate; Creatinine. |

History of valvuloplasty refers to balloon aortic valvuloplasty prior to the TAVR procedure.

ECMO = extracorporeal membrane oxygenation; THV = transcatheter heart valve; CT = computed tomography; LAA = left atrial appendage; PFO = patent foramen ovale; STS = Society of Thoracic Surgeons; TAVR = transcatheter aortic valve replacement; SAVR = surgical aortic valve replacement; DLZ = device landing zone.

**Supplementary Figure 1. Precision-recall curve for the choice of decision threshold value**

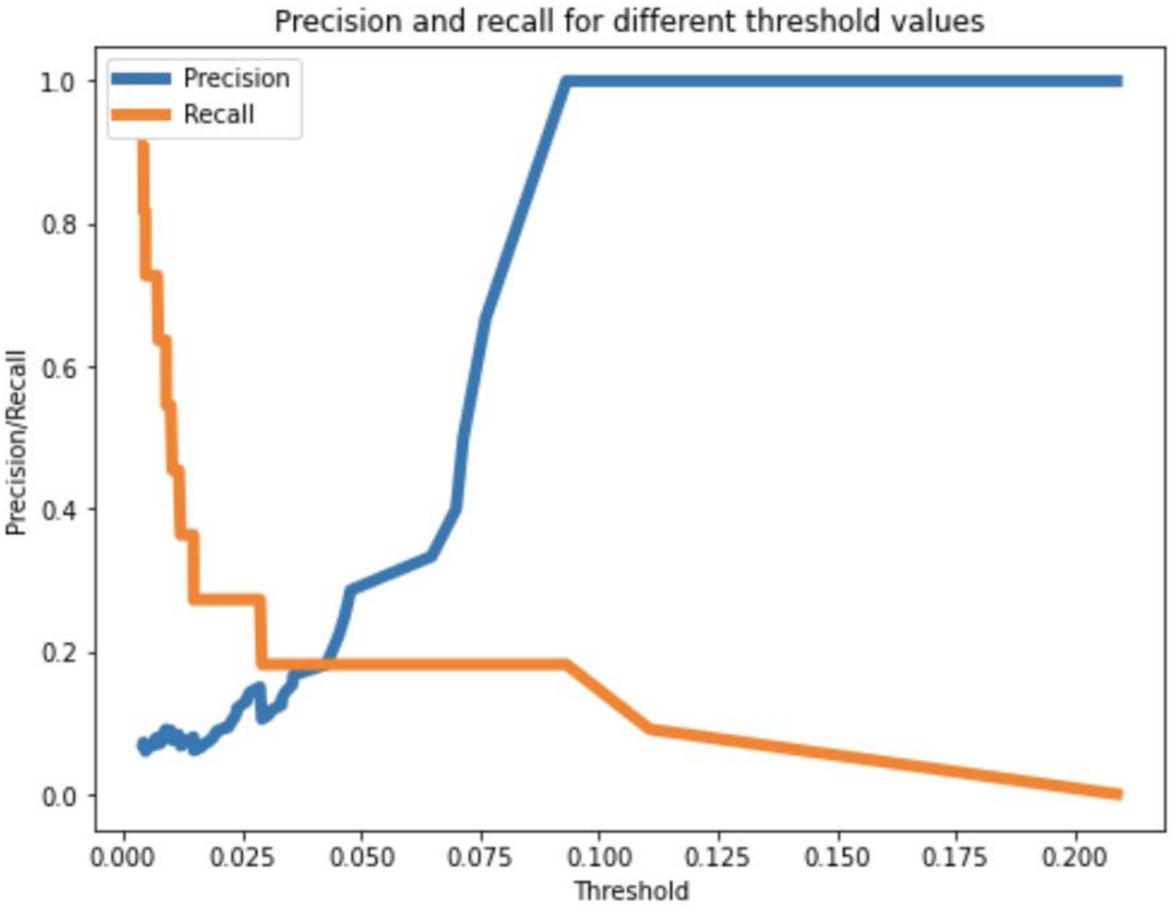


The chosen threshold was 0.01.

**Supplementary Figure 2. Top 20 variables classified by importance for the predictive model**

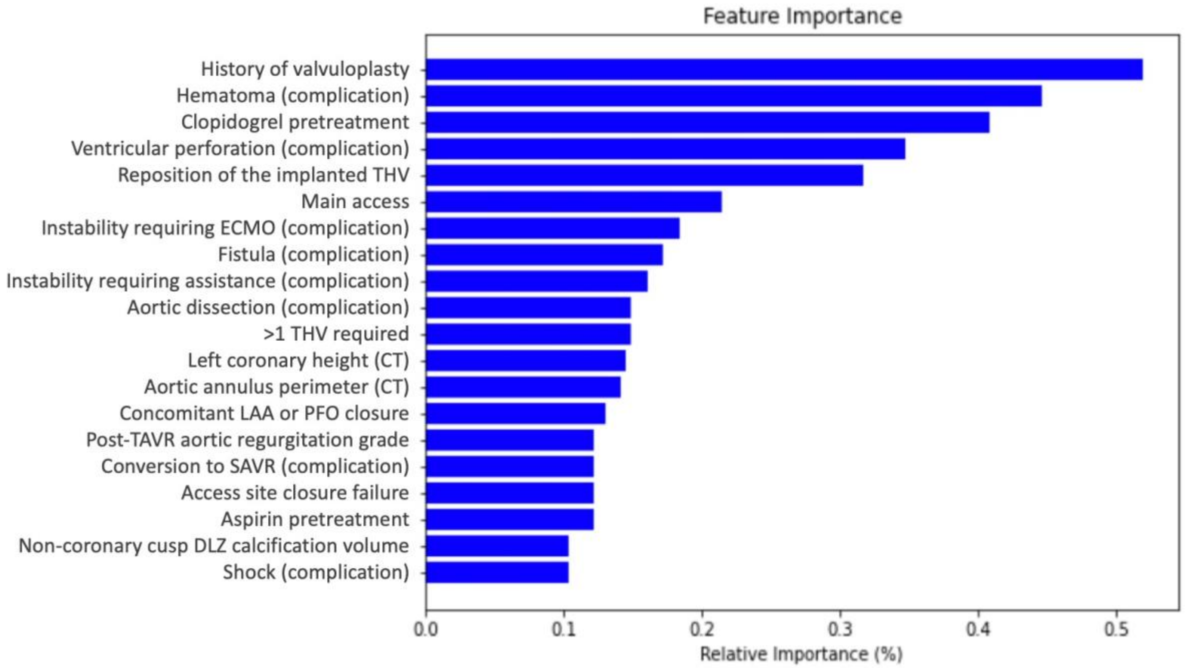


History of valvuloplasty refers to balloon aortic valvuloplasty prior to the TAVR procedure.

ECMO = extracorporeal membrane oxygenation; THV = transcatheter heart valve; CT = computed tomography; LAA = left atrial appendage; PFO = patent foramen ovale; TAVR = transcatheter aortic valve replacement; SAVR = surgical aortic valve replacement; DLZ = device landing zone.
